# Behavioural manifestations of human-directed social motivation in dogs

**DOI:** 10.1038/s41598-025-34929-w

**Published:** 2026-01-19

**Authors:** Mónica Boada, María Victoria Hernández-Lloreda, Fernando Colmenares, Josep Call

**Affiliations:** 1 Grupo UCM de Psicobiología Social, Evolutiva y Comparada, Departamento de Psicobiología y Metodología en Ciencias del Comportamiento, Facultad de Psicología, Campus de Somosaguas, Universidad Complutense de Madrid, Madrid, 28223 Spain; 2 School of Psychology and Neuroscience, University of St Andrews, St Andrews, KY16 9JP UK; 3 Global Research Centre for Diverse Intelligences, University of St Andrews, St Andrews, KY16 9AJ UK

**Keywords:** Social motivation, Sociability, Human-dog relationship, Dog domestication, Psychology, Animal behaviour

## Abstract

During domestication, dogs might have been selected for an increased motivation to engage socially with humans. A more nuanced conceptualization and evaluation of social motivation can improve our understanding of how domestication shaped dogs’ psychology. We tested 104 dogs in a battery that assessed behaviours used as indicators of three aspects of social motivation: social orienting, reward, and maintaining. We aimed to test whether these behaviours indeed represented manifestations of the same construct, in which case we predicted a positive correlation between them. We found few significant correlations, some going in the opposite direction of our predictions. Our results might imply that the tests measured different traits, or that examined behaviours were not driven by the same motivational mechanism. Alternatively, the tests did not reveal sufficient individual variability or provide a clean measure, preventing us from finding existing associations. Moreover, it is possible that, contrary to our assumptions, social motivation is not consistent across contexts and is a context-specific trait instead. Future research should determine how to measure dogs’ social motivation validly and reliably, re-examine assumptions regarding the motivational mechanisms underlying behaviours in commonly used tests and investigate interindividual differences in contextual plasticity regarding behavioural manifestations of social motivation.

**Supplementary Information:**

The online version contains supplementary material available at 10.1038/s41598-025-34929-w.

## Introduction

 Tameness is one of the central features of the domestication syndrome (DS)^1^ and can be seen as a phenotype composed of multiple traits, including not only low fearfulness and aggression but high social motivation towards humans. In dogs specifically, human-oriented social motivation (i.e., a propensity to seek proximity and engage socially with humans) has been hypothesized to be a fundamental target of selection during the domestication process. For example, the *hypersociability hypothesis* postulates that domestication resulted in dogs showing hypersocial responses towards humans compared with wolves^2^. A more nuanced conceptualization and evaluation of the social motivation construct can therefore be valuable for gaining a better understanding of the changes that dogs’ psychology has undergone during the domestication process (for a review see ref.^3^). A conceptual framework that could be useful for the study of human-directed social motivation in dogs is that devised by Chevallier et al.^4^ in the context of the social motivation theory of autism (see ref.^5^ for a similar view). They proposed that, in humans, there are three categories of behavioural manifestations of social motivation: (1) social orienting, (2) social reward, and (3) social maintaining.

Social orienting refers to attentional priority granted to social signals or objects with social importance^4^. The authors highlight how our attention is easily captured by human faces and how individuals with autism spectrum disorder (ASD) show a reduced tendency to establish eye contact. The human face is a stimulus of high social relevance not only for humans but also for dogs. Dogs obtain important social information from human faces, such as identity and emotional states, and their gazing behaviour shares some similarities with that of primates (e.g., attentional focus on the eyes)^6^. Thus, studying gazing behaviour towards humans could be a suitable approach to assess this aspect of social motivation in dogs. Dogs have a spontaneous tendency to gaze at human faces from a young age, in contrast to wolves (e.g., ref.^7^), but individual variation and breed differences exist^8,9^. Dogs resort to us when confronted with problems they are unable to solve by themselves (e.g., refs.^9–16^), and gazing behaviour towards humans has often been interpreted as a help-requesting behaviour (but see refs.^17–19^). In this context, the proportion of time dogs spend looking at a human instead of at the apparatus involved in a problem-solving task has been taken as a measure of attentional bias to social stimuli^2^.

The second aspect of social motivation is finding social interactions rewarding (i.e., social reward)^4^. Potential deficits in this aspect, found in individuals with ASD, include a diminished response to social rewards (e.g., verbal praise) and reduced preference for collaborative activities. Regarding dogs’ responses to social rewards, previous studies suggest that there might be a high degree of individual variability in the preference between social (petting or verbal praise) and food rewards^20–22^. Concerning dogs’ preference for collaborative activities, dogs are able to solve cooperative problems with a human partner (e.g. ref.^23^) and recruit a partner when needed^24^. Yet, to our knowledge, whether dogs choose to cooperate with a human partner or to solve a task individually was only investigated recently^25^. However, the study was based on a stag hunt game, in which the human partner chose first whether to cooperate (stag) or solve the task individually (hare), and dogs had to adjust their behaviour accordingly to maximize their payoff. In the control group, where choosing either stag or hare yielded the same reward for the dog when the human chose stag, dogs showed no preference for cooperation. A more naturalistic situation that requires cooperation is social play, which involves behavioural coordination between the partners^26^. Studying whether dogs show a preference for social play with a human over individual play, as has been shown in non-human apes^27^, might provide some insight regarding their preference for collaborative activities. Recently, Horschler et al.^28^ revealed that dogs attempt to re-engage a human partner when joint play is interrupted. Given that dogs could play on their own during the interruption period, a possible interpretation of the re-engagement attempts is that dogs found social play more rewarding than individual play. However, as the study was not designed to compare social and individual play, the latter was not measured.

The third aspect of social motivation refers to efforts dedicated to fostering and maintaining social bonds^4^. This includes so-called maintaining strategies, which are behaviours intended to establish, maintain and enhance social relationships. Among these we find ingratiating behaviours, which increase an individual’s likeability. A behaviour that fulfils this function without the individual’s conscious awareness is nonconscious mimicry (i.e. chameleon effect)^29^. Relatedly, it has been found that dogs synchronize their behaviours with that of humans in a variety of situations. This dog-human behavioural synchronization depends on the degree of affiliation between the partners, and is suggested to act as a social glue (see ref.^30^ for a review). Also related to the chameleon effect is the concept of conformity, which implies “changing one’s behaviour to match the responses of others” (ref.^31^, p. 606]). In this sense, several studies have shown that dogs conform to choices made by humans, ignoring their personal preferences, even if it is counterproductive, when presented with human communicative cues^32–34^.

If the three categories of behaviours considered earlier are manifestations of a single underlying construct or, in other words, if they share the same underlying motivational mechanism, they should be correlated. Few studies have examined the association between these potential behavioural indicators of social motivation in dogs, and so far there is limited evidence of a correlation between them^35–39^. In a factor analysis, a social referencing task that measured dogs’ tendency to gaze at a human when social play was interrupted, which could be an indicator of social reward, grouped together with an unsolvable task that measured dogs’ tendency to look at the human during the unsolvable trials, which could be taken as a measure of social orienting^36^. Positive correlations have been found between efficacy of social reinforcers and behaviours in a reunion test^37^, both of which could be indicators of social reward. Oxytocin administration influenced both the duration of proximity in a synchrony test and the gaze duration at the caregiver in a shared attention test^39^, which provides indirect evidence of a link between social maintaining and social orienting.

The objective of our study was to gain a better understanding of dogs’ human-oriented social motivation, a potential key element in the domestication process. We measured different social behaviours in pet dogs, conceptually organized around three categories: social orienting, reward and maintaining. We aimed to test whether these behaviours indeed represented manifestations of the same construct, in which case we predicted we would find a positive correlation between them.

## Methods

### Ethical statement

All methods were performed in accordance with the relevant guidelines and regulations. The Animal Experimentation Committee of the Complutense University of Madrid (UCM) deemed that an ethics approval was not required given that the procedures were non-invasive and dog subjects were never forced to participate. Regarding human participants, given the minimal involvement of the dog caregivers and the non-invasive nature of the experimental protocols, ethical approval from a specific institutional and/or licensing committee was not obtained. At the time of the study, the Research Ethics Committee of the UCM, which is responsible for evaluating projects involving interventions with or on human beings, the use of human biological samples, or the use of personal data, had not yet been established. Written informed consent was obtained from all dog caregivers for their participation and that of their dogs in the study. As outlined in the informed consent, all personal data provided by the participants has been treated with the utmost confidentiality and in full compliance with Regulation (EU) 2016/679 and other applicable regulations. All methods are reported in accordance with the ARRIVE guidelines.

### Subjects

The sample consisted of pet dogs which were recruited from individuals, by word of mouth and through social networks. Participants were also recruited among the students of the Psychology Faculty of the UCM. The inclusion criteria comprised characteristics of both the dog participants and the homes. Dogs with vision or hearing impairments were considered ineligible, and participation was dependent on caregivers’ assessment of their dog’s compliance with these criteria. Dogs also had to be at least one year old to participate, since it has been observed that personality consistency in general is higher in adults, and no sociability consistency has been found in pups^40^. In addition, large changes in behaviours related to communication and social motivation from early ontogeny to young adulthood have been observed in dogs^41^. Only residents in the Community of Madrid that could offer a space of approximately 3 × 2 m could participate.

A total of 104 dogs participated in the study, out of which 53 (51%) were male (neutered: 34, intact: 19) and 51 (49%) female (spayed: 41, intact: 10). The mean age was 5.7 years (*SD* = 3.3; range: 1–14); for 30 dogs (29%) the age indicated by the caregiver was an estimation. Fifty-one dogs (49%) were classified by their caregivers as purebreds and 53 (51%) as mixed-breed (see Supplementary Table S1 for full demographic information). At the time of enrolment, most dogs had been with their current family for years (92.3% for at least one year; *M* = 57.9 months; *SD =* 37; range: 2-156) and had joined the family during puppyhood (72.1% when they were 6 months old or younger; *Mdn* = 2 months; *Q1-Q3 =* 0-9.5). Three dogs were not tested in the second session due to scheduling constraints with their caregivers.

### Design

**Fig. 1:**
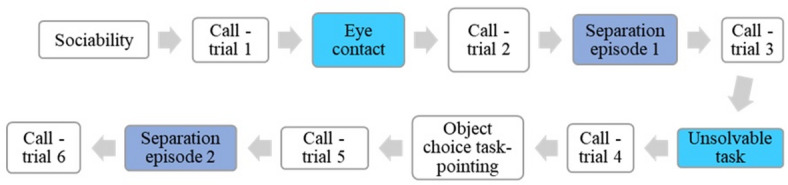
Presentation order of the tests in the first session of the study. Social motivation tests, discussed in this article, are marked in colour (different colour for each category; light blue: social orienting, dark blue: social reward).

**Fig. 2:**
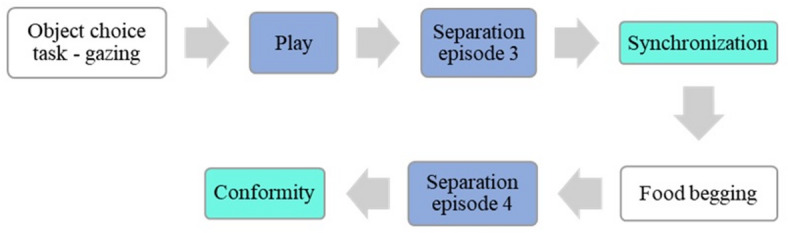
**Presentation order of the tests in the second session of the study.** Social motivation tests, discussed in this article, are marked in colour (different colour for each category; dark blue: social reward, aquamarine: social maintaining).

To examine the different behavioural manifestations of social motivation, a behavioural test battery was used. The battery included tests that measured sociability and social cognition as well, which will not be discussed in the present article. The battery consisted of 11 tests in total, out of which six were used to measure the three categories of behavioural manifestations of social motivation (two tests per category). The tests were always run in the same order. Although performing the tests in the same order with all the subjects does not allow to remove potential order effects, it makes comparison between individuals possible (e.g., refs.^35,36,38^). The tests were spread out over two sessions which were carried out on separate days in the caregivers’ homes, with the inter-session period being variable due to circumstances such as the caregiver’s availability and restrictions derived from the public health alarm situation (COVID-19 pandemic). The mean inter-session period was 16.2 days (*SD =* 10.6; range = 1–67). Each session had an approximate duration of 60–75 min, including breaks between tests for setting up and explaining protocols to the caregivers (see Figs. 1 and 2 for the tests presented in each session).

### Materials

**Fig. 3:**
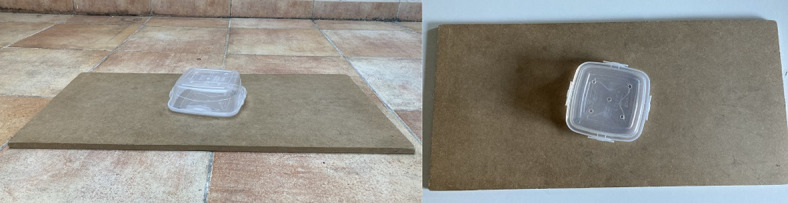
**Apparatus for the unsolvable task.**

The food rewards used were pieces of sausage, unless the subject had some dietary restriction, or the caregiver thought that other type of food could be more motivating for their dog. For the *unsolvable* task, we used an apparatus consisting of a plastic container placed upside down on a wooden board (59.5 × 30 × 1 cm) (Fig. 3). The lid of the container was fixed to the board, so when the container was closed with the lid, it could not be separated from the board. During the study the container was broken and had to be replaced, so two types were used (container 1: 14 × 11 × 5.5 cm; container 2: 11 × 11 × 4.5 cm). The container had several holes in its bottom part, which was facing up, so the subject could smell the food reward. In the *play test*, two types of toys were used: rope bone (type A) and ball with rope (type B). Since some toys had to be replaced during the study, but the same model could not be found, the same toys were not used for all the subjects (but they were of the same types). In each trial the two toys were always identical. For the *conformity test*, the transparent lids of some plastic containers, of 12.5 cm diameter, were used as plates.

### Procedure

The caregivers were asked not to feed their dog in the 4 h before the test session, and to give them a walk before the session or try to avoid having the session during times associated with walks. For the first session, they were told that the dog should not see the experimenter before the first test. In the cases in which the caregiver did not follow this indication, the experimenter made sure that contact with the subject was minimum and asked the caregiver to take the dog to a different room until the first test was initiated.

**Fig. 4:**
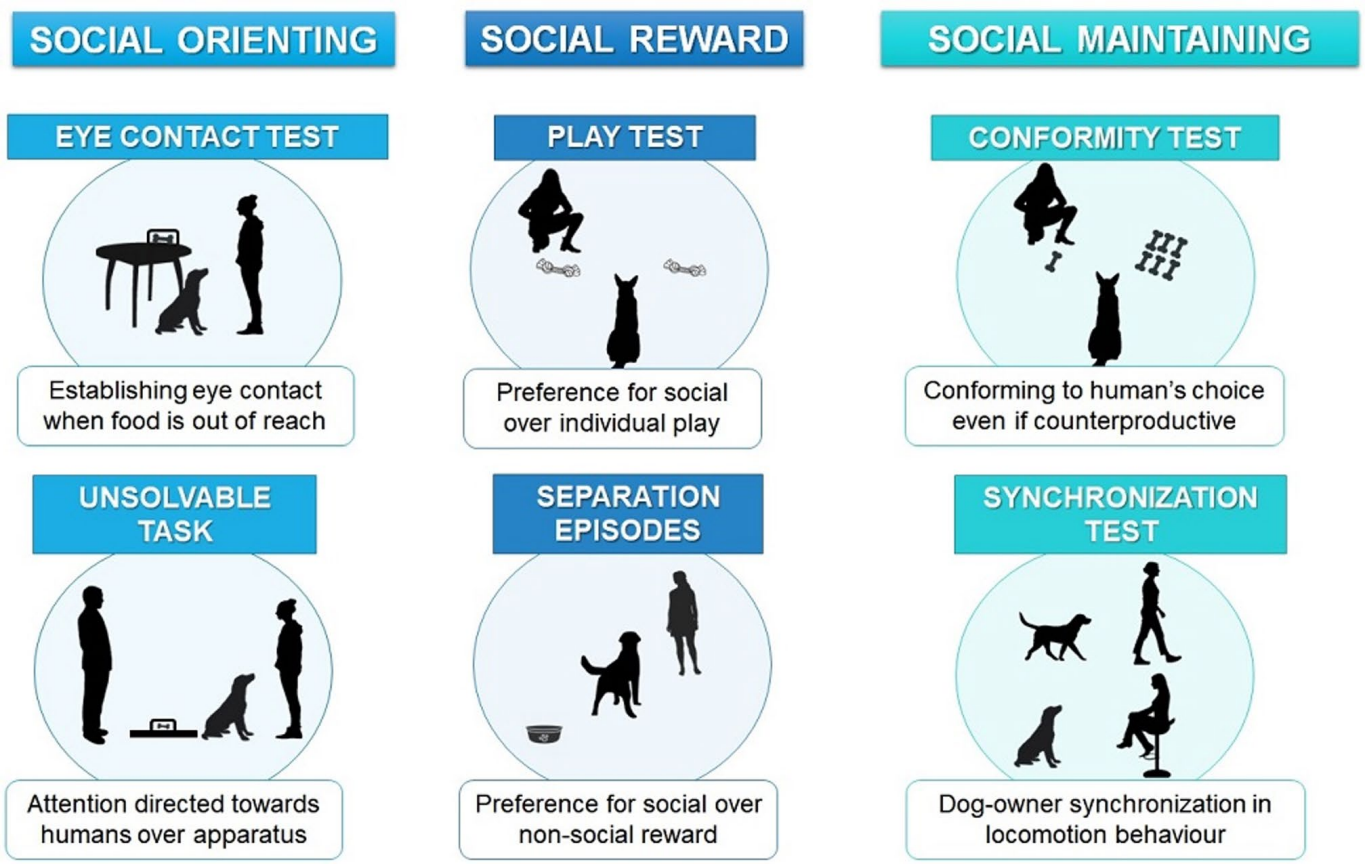
**Behavioural tests for the assessment of social motivation.** Representation of the tests used to measure the three categories of behavioural manifestations of social motivation. See the text for full descriptions of the procedure of each test.

Below we briefly describe the social motivation tests, which are classified according to the category they were intended to measure (see also Fig. 4). Detailed descriptions of these tasks, as well as short descriptions of the other tests that made up the battery are included in the Supplementary Methods.

### Social orienting

*Eye contact test.* The goal of this test, based on the protocol in ref.^42^, was to measure dogs’ tendency to establish eye contact when food was out of reach. The test consisted of two phases of 1 min each. In the first phase, the experimenter gave the subject pieces of food from a container placed on an elevated surface out of reach of the animal. In the second phase, the experimenter gave them one final piece of food and gazed at the subject. The coded variables were the latency to gaze at the experimenter and the gaze duration after the last piece of food was given.

*Unsolvable task*. The goal of this test, based on protocols in refs.^13,43^, was to measure dogs’ tendency to direct their attention towards humans over an apparatus containing a food reward. The experimenter and the caregiver stood on opposite sides of the apparatus, with the caregiver holding the subject, while the experimenter baited the apparatus. The test began with three solvable trials, in which the subject could obtain the food reward from the apparatus. The trial ended when the subject obtained the food or after 1 min. If the subject obtained the food in at least two out of three trials, a final trial was carried out, which was unsolvable and lasted 1 min. The coded behaviours were gazing at the apparatus and at humans (caregiver or experimenter) (i.e., turning/lifting head towards the apparatus/human from a stationary position) (latency and duration), interacting with the apparatus (i.e., directing a behaviour towards the apparatus such as licking, sniffing, scratching) and with humans (i.e., establishing physical contact with caregiver or experimenter) (latency and duration) and alternating gaze between apparatus and humans (frequency and latency). Since the test was re-coded considering the unsolvable trial started when the subject first interacted with the apparatus, each subject had a different test duration, which was used to calculate relative durations and latencies (see Supplementary Information).

### Social reward

*Separation episodes.* The goal of this test, adapted from ref.^22^, was to assess dogs’ preference for a social reward over a food reward. Four separation episodes were carried out, in which the caregiver left the house for 1 min. At the end of the minute, the subject could choose between greeting the caregiver – who could greet the dog as they normally would, once the dog was within arm’s reach – or eating their favourite food reward from a bowl. The separation episodes were spread over the two sessions (two episodes in each session) and were interspersed with the rest of the tests (see Figs. 1 and 2). We initially recorded only the first choice but given that after an initial group of subjects was tested it was observed that there would probably be low variability (i.e., most subjects chose the food reward), we decided to examine the number of episodes in which they approached the caregiver after choosing the food reward. To this end, the experimenter waited up to 30 s or until the subject left the test zone or stayed in front of the experimenter ignoring the caregiver.

*Play test.* The goal of this test, based on protocols in refs.^27,44^, was to measure dogs’ preference for social over individual play. At the start of each trial, the experimenter showed two identical toys to the subject and placed both toys on the floor simultaneously. Next, she kneeled behind one of them and gave the indication to the caregiver to release the subject. The trial lasted 1 min, during which the subject was free to choose whether to play or not and with which toy. The experimenter remained looking at the toy in front of her and only if the subject approached her (at arm’s reach) invited the subject to play, moving the toy on the floor and talking (dog-directed speech). If the subject grabbed the toy, the experimenter played with them grabbing the other end of the toy. If the subject was playing with the experimenter when the minute ended, the experimenter suddenly let go of the toy, interrupting the game. The interruption period lasted 1 min, during which the experimenter remained passive. Four trials were run; in the first two trials the toys used were rope bones, and in the last two they were balls with a rope. The side of the toy behind which the experimenter kneeled was counterbalanced between trials and pair of toys. The order was the same for all the subjects (except one, due to an experimenter’s error). The coded variables were the first toy approached (within 0.5 m approximately), the first toy touched, the duration of proximity to the experimenter (i.e., any body part within arm’s reach of the experimenter − 65 cm approximately-) and the duration of interaction with each toy (which included rubbing, nosing, licking, sniffing, pawing, chewing, holding in mouth and following the toy’s movement with the head, but excluded physical contact without active interaction such as lying on the toy). Behaviours during the interruption period were coded but not analysed in this study.

### Social maintaining

*Synchronization test.* The goal of this test, adapted from ref.^45^ was to measure dogs’ tendency to synchronize their locomotor behaviour with their caregiver. In this test, the caregiver took a route previously set by the experimenter, in which they did the following: (1) stand up from a chair in a room and walk during 15 s towards a second room, (2) at the end of the 15 s, sit on a chair located on the second room and remain sitting for 15 s, (3) stand up and walk during 15 s back to the first room, (4) at the end of the 15 s, sit on the chair where the test had started and remain sitting for 15 s. Thus, the test consisted of four phases of 15 s each, i.e., it lasted for 1 min. During the test the caregiver was instructed to ignore the subject, who was free to move wherever they wanted. The main variables coded were the duration of synchronized activity (subject and caregiver are simultaneously moving/stationary) and the latency to switch to the same activity as the caregiver.

*Conformity test.* The goal of this test, based on protocols in refs.^32–34^, was to measure dogs’ social susceptibility when choosing between two plates baited with different amounts of food (small amount: one piece; large amount: six pieces). A pre-test was conducted first, in which the subject could choose between the two plates without social influence. The pre-test consisted of four to six trials (see Supplementary Methods for an explanation of the criterion to move on to the test phase) and the goal was to check whether the subject showed a preference for the large amount. In the test phase, the experimenter showed interest for the plate with the small quantity by grabbing the piece of food, bringing it close to her face, alternating gaze between food and subject and saying in Spanish with a happy tone “How tasty! How tasty this is!”. This phase consisted of six trials. In both phases the subject was allowed to eat the food from the chosen plate. The position of the plates was counterbalanced, with the restriction that the same position could not be repeated in more than two consecutive trials. The order of the trials was the same for all subjects. The main variable coded was the plate chosen.

### Data coding and reliability

In each test, variables were recorded during the experiment or coded from video. Some of the variables that were initially coded were later discarded in the data pre-processing phase due to a variety of reasons and were not used in subsequent analyses. Furthermore, in some cases the decision was made to code the variable differently, create a new variable, or to keep only subjects that fulfil certain criteria. These decisions and an experimenter’s error affecting data from the conformity test are explained in the Supplementary Methods. The description and final sample size of the retained variables can be found in Tables 1, 2 and 3. For descriptive statistics see Supplementary Tables S2 and S3.

Videos were coded in Solomon Coder (beta 19.08.02, © András Péter) with a time resolution of 0.2 s. All videos were coded by the first author (M.B.), and a randomly selected 20% of videos were coded by two persons not involved in the study to calculate interobserver reliability (assistant A: eye contact test, unsolvable task, synchronization test; assistant B: separation episodes, play test, play test – interruption period). The variables of interest of the conformity test were considered to have been coded unequivocally, so calculating the interobserver reliability was not deemed necessary. For continuous variables the intraclass correlation coefficient (ICC) estimates and their 95% confidence intervals were calculated using the *icc* function from *irr* package in RStudio^46^, based on single-rating, absolute-agreement, two-way random effects model. For categorical variables, Cohen’s kappa was calculated using the *kappa2* function from *irr* package in R. ICC and kappa values of the relevant variables can be found in Supplementary Table S4. All variables except latency to gaze at humans and frequency of and latency to gaze alternation in the unsolvable task had ICC ≥ 0.8 and kappa ≥ 0.7.

### Statistical analyses

Statistical analyses were conducted in IBM SPSS Statistics version 27, jamovi version 2.3.18^47^ and RStudio version 2023.3.1.446^48^ (the choice of statistical software reflected the analysts’ familiarity and preferences). All tests were two-tailed and statistical significance was tested at the 0.05 level.

The Kendall correlation coefficient was used for ordinal variables, as well as for continuous variables, since they did not follow a normal distribution (Shapiro-Wilk test, *p* < 0.05) and some of them were truncated. Point and rank biserial correlations were calculated for the association between binary and continuous variables, and binary and ordinal variables, respectively.

## Results

**Fig. 5:**
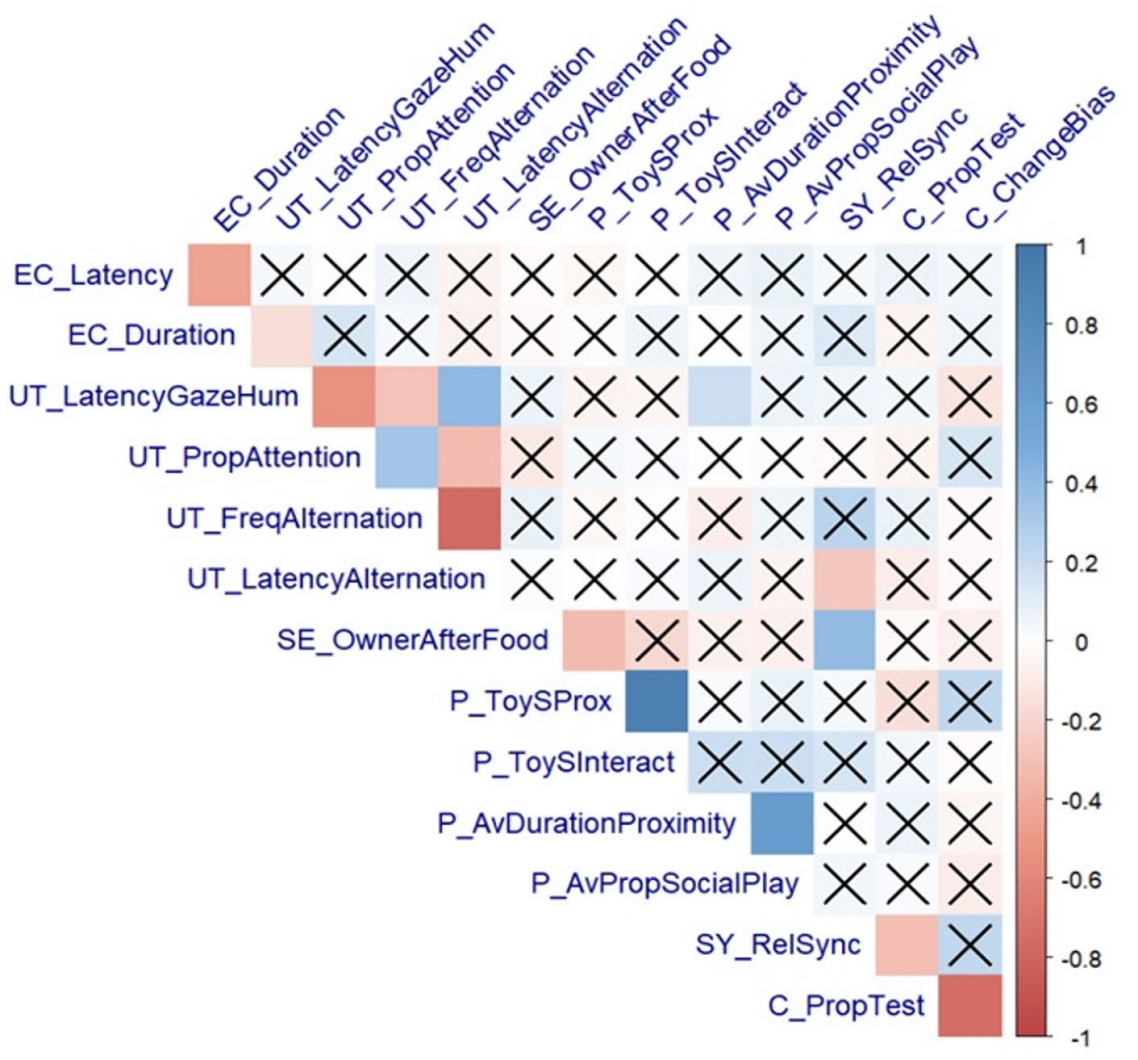
**Correlation matrix of the social motivation tests.** Non-significant correlations (*p* > 0.05) are crossed. The direction of the correlation is indicated by the color (blue: positive, red: negative) and the strength is indicated by the color gradient. EC = eye contact test; UT = unsolvable task; P = play test; SE = separation episodes; SY = synchronization; C = conformity.

Only statistically significant correlations are reported here (for all correlations see Supplementary Table S5). As can be seen in the correlation matrix (Fig. 5), most significant correlations and those with the highest values were between variables within the same test. Few correlations between tests reached statistical significance and some correlations went in the opposite direction to our predictions (Table 4).

## Discussion

In this study, we tested pet dogs in tasks that we considered could assess the three categories of behavioural manifestations of social motivation defined by Chevallier et al.^4^: social orienting, social reward and social maintaining. This involved choosing social behaviours in dogs that could be functionally analogue to the human behaviours that the authors mentioned in their proposal (see also ref.^5^). Arguably, behaviours that are measuring the same construct or are controlled by the same motivational system should correlate with each other (i.e., convergent validity). However, the pattern of correlations we found provided little empirical support for convergent validity across the social behaviours examined. Only a few significant correlations between tests emerged (6 out of 90) and some went in the opposite direction to what we predicted (Table 4). Our results are in line with previous studies with dogs in which putative social motivation variables have failed to group together in structural analyses^35,37^, but stand in contrast with others that provided suggestive evidence that social behaviours that could be conceptualized as social motivation indicators might indeed be measuring the same construct^36,39^.

Several of the correlations we found were consistent with our expectations. Dogs that established eye contact for a longer duration during the eye contact (EC) test had a shorter latency to gaze at the humans during the unsolvable task (UT). Since in both tests there was a food reward that was unobtainable and a human was present, these variables could be taken as indicators of dogs’ leaning towards human-oriented social strategies when facing a problem they are unable to solve by themselves. Moreover, dogs that were more synchronized with their caregiver in terms of their activity were faster to alternate gaze between the apparatus and the humans during the UT. This suggests that dog-caregiver behavioural synchronization is related to communicative behaviours emitted during a problem-solving task. We also found that dogs that approached the caregiver more often (after choosing food) during reunion in the SE were more synchronized with their caregiver. Apart from measuring social motivation, both variables might be related to the dog-caregiver bond. This association is in line with the very weak to weak positive correlations observed in the correlation matrices of ref.^37^ between synchronicity and orienting behaviour during a reunion test. Lastly, dogs that were more synchronized with their caregiver chose the large quantity less often in the conformity test, which means that they chose the plate favoured by the experimenter more frequently. Although this could suggest that there is a link between the degree of dog-caregiver behavioural synchronization and dogs’ tendency to conform to human choices, this analysis included subjects that did not show a preference for the large quantity in the pre-test (i.e., did not reach criterion; see Supplementary Information). Conducting the analysis only with subjects that reached criterion, the correlation coefficient was slightly higher, but was no longer statistically significant, as expected given the reduced sample size (*n* = 17, Kendall’s Tau = − 0.35, *p* = 0.063). Moreover, we found no correlation with the change-in-bias, which measured the difference between subjects’ choices in the pre-test and the test, and intended to capture dogs’ social susceptibility.

A couple of the correlations we found went in the opposite direction to our predictions. Dogs that took longer to gaze at humans during the UT, spent more time in proximity to the experimenter during the play test. However, they did not necessarily play more with the experimenter, since no association was found with the average proportion of social play. The correlation we found might be mediated by dogs’ food motivation. Since the play test took place after one condition of the object choice task (OCT), which had a similar setting but involved food, some dogs that approached the experimenter during the play test might have been trying to obtain food. These subjects would also have been more focused on obtaining the food during the UT, hence taking longer to gaze at the humans. To some degree, this negative association between gazing behaviour during the UT and proximity to the experimenter during the play test is in line with results of ref.^38^ but stands in contrast to findings of ref.^36^. In the study by Turcsán and collaborators^38^, there was a negative relationship between gaze duration towards humans in a problem-solving test and gaze alternations between ball and caregiver in a ball play test. In the study by MacLean and coworkers^36^, in contrast, gaze duration in an UT and after the interruption of a social game both loaded positively onto the same factor. To make a more direct comparison, we checked whether the duration of gazing at the experimenter during the interruption period of our play test was associated with variables from the UT. Keeping only subjects that had data on gazing behaviour during four interruption trials in the play test, we did not find any significant correlation with variables from the UT. Nevertheless, the sample size was small (*n* = 11–12) and duration of gazing during the interruption period was low overall. Finally, we found that dogs that approached the caregiver more often after separation (after choosing food) were less likely to approach the social toy first, which does not fit with our view that both variables were indicators of social reward. A potential explanation is that dogs that approached the caregiver more often were less willing to stay near the experimenter during the separation episodes (SE) and during the play test. This seems unlikely and is unsupported given the lack of correlation between the frequency of approaching the caregiver after separation and the duration of proximity to the experimenter during the play test. Another possibility, if we suppose as aforementioned that subjects approaching the experimenter during the play test might have been trying to obtain food, is that subjects that approached the caregiver less often after separation and were more likely to approach the social toy first were primarily driven by food motivation. Thus, after obtaining the food reward in the separation episodes, they showed little interest in greeting the caregiver, and in the play test they approached the social toy more often because they expected food from the experimenter.

It is important to note at this point that failing to find correlations between behaviours that are theoretically measuring the same construct is not uncommon. For example, several studies in dogs have revealed that scores on inhibitory control tasks are not correlated at all or only weakly^49–53^ (for a cross-species review see ref.^54^). When weak or no correlations across tasks purported to measure inhibitory control have been found, an often-reached conclusion is that the construct is multidimensional, and tasks are measuring different dimensions. Following the model of Chevallier et al.^4^, social motivation could be seen as a multidimensional construct with, at least, three dimensions: social orienting, social reward and social maintaining. Thus, the lack of behavioural correlations between tests categorized as belonging to potentially different social motivation dimensions should not necessarily make us conclude that they are not measuring the same construct. Yet, we did not find substantial evidence of correlation between behaviours within the same dimension either (only 3/15 correlations were significant, excluding correlations within tests). This is in line with findings from studies of components of inhibitory control in dogs and of executive functions in humans^49,50,55,56^. Admittedly, social motivation can be an imprecise construct^57,58^, alternative categorizations of the social motivation construct may well be possible (e.g.,^58^), and even when proposing specific dimensions, the boundary between them can be fuzzy (classification of behaviours into one of the three dimensions of social motivation in our study is arguable). Another factor that might have affected our results is that the human involved in the tests differed (experimenter, caregiver or both). Although familiarity is known to influence human-directed behaviour in dogs, this study was aimed at exploring social motivation at a more general level, using a framework that sought to examine dogs’ behavioural responses across different contexts. Using an experimenter to carry out the tests helps maintain standardization and ensure strict adherence to testing protocols. The caregiver was only involved in tests that we thought would not elicit the behavioural responses of interest otherwise: the separation episodes and the synchronization test. In any case, it is important to note that we did find associations between behavioural responses in tests involving different humans. An additional consideration is that even if behaviours in different tests share the same motivational mechanism, they might be uncorrelated if they depend on other factors apart from social motivation (i.e., “task impurity problem”). For example, behaviours in the play test might have also been influenced by food expectation or the movement of the toy held by the experimenter. On one hand, subjects generally spent more time playing individually than gazing at the human during the interruption periods, which suggests that they were mainly interested in the toy. On the other hand, they also showed re-engagement behaviours, such as establishing physical contact with the experimenter, vocalizing, or giving the toy (i.e., leaving it close to the experimenter and then staring at it, alternating gaze, vocalizing, etc.), which indicate that dogs were motivated to engage in social play with the human. Finally, how we operationalized some aspects of social motivation in this study implied assessing preferences between social and non-social stimuli (e.g., social orienting referred to preferential attention towards social stimuli, and social reward alluded to a preference for social rewards and activities). Consequently, it could be argued that some of our tests do not directly assess the strength of social motivation but rather indicate the difference in motivation strength between the stimuli presented.

Importantly, in our study we assumed that social motivation is a construct that should show consistency across contexts. However, the lack of correlation between behavioural tests of social motivation could be interpreted as evidence that the trait is context-specific. Consistency across contexts is affected by the existence of individual behaviour profiles^59^. If individuals show different behavioural response profiles or, in other words, if individuals vary in their contextual plasticity, variable-oriented correlations cannot be strong. We conducted cluster and profile analyses and found some evidence of the existence of different profile types, some of which showed consistently high social motivation across contexts (low contextual plasticity), while others varied their expression of social motivation depending on the context (high contextual plasticity)^60^. However, when comparing cluster solutions with those provided by latent profile analysis, the composition of the clusters/profiles was sometimes not equivalent. Moreover, the clusters/profiles often showed no association with conceptually related and/or demographic variables. Thus, the validity and utility of the cluster solutions we found seemed limited.

Our study has various limitations that restrict the conclusions we can derive from the findings reported. A set of limitations is related to the psychometric properties of the tests in our battery, particularly their reliability and the individual variability that they could reveal. Unreliable data could attenuate observed correlations^61^. One of the limitations of our study is that we did not evaluate test-retest reliability, which is known to affect phenotypic correlations^62,63^. In addition to this, some tests might not have been adequate to reveal between-individual variation, as evidenced by skewed distributions and possible floor and ceiling effects. For instance, in the unsolvable task, the proportion of attention towards humans was right-skewed, which might imply that a longer test duration was necessary (e.g., ref.^64^), since many subjects spent most of the test attending to (i.e., gazing at/interacting with) the apparatus (see also refs.^17,18,65^). Tasks that produce robust experimental effects, and hence become popular, often show low between-subject variability, making them less suitable for correlational studies (i.e., “the reliability paradox”^66^). An issue we have not commented on is that if during domestication dogs were consistently selected for high social motivation, as proposed by various hypotheses (e.g., *emotional reactivity hypothesis*^67,68^, *hypersociability hypothesis*^2^), this might have reduced their variability on this trait. Indeed, in some studies wolves show higher variability in human-directed sociability than dogs^21,69^ (for a review, see ref.^3^). This potentially reduced variability means that we might need to make a greater effort to uncover individual variation in social motivation in dogs. In addition, sample size might have been a limiting factor in the case of some bivariate associations. The use of a fixed test order and number of sessions might have also affected the results due to carry-over effects and increased fatigue towards the end of the sessions. As a final note, although the tests were standardized as much as possible, the characteristics of each home were different, and the experimenter had limited control over the testing environment. Testing dogs in a laboratory could therefore be valuable and would enable conducting some tests that are not feasible at the caregivers’ homes due to apparatus or instruments needed.

Future studies should determine what tests can be used to measure different aspects of social motivation in dogs validly and reliably and re-examine the assumptions regarding the motivational mechanisms that might be driving behaviours in commonly used tests. Combining behavioural studies with analysis of physiological (e.g., hormones such as oxytocin) and neurological (e.g., caudate activation) measures is desirable. We believe it is important to study interindividual differences in social motivation and their consistency across time. It is also relevant to determine whether social motivation should be regarded as a context specific trait rather than expecting consistency across behavioural manifestations of social motivation in different contexts. Taking an individual-oriented approach allows to unveil individual profiles of behavioural responses across contexts and can be used to identify clusters of individuals with similar profiles. Future research can investigate interindividual differences in contextual plasticity with regards to behavioural manifestations of social motivation.

## Conclusion

This study found few significant behavioural correlations between tests selected to assess three aspects of human-directed social motivation in dogs: social orienting, social reward and social maintaining. The scarcity of correlations across putative social motivation tests might imply that these tasks measured different traits, or that the behaviours expressed in these tests were not driven by a shared underlying motivational mechanism. Alternatively, the studied behaviours might still be valid indicators of social motivation, but the tests might have been inadequate for the study of individual differences or might not provide a clean measure, preventing us from finding existing associations. Another possibility is that, contrary to our assumptions, social motivation is not consistent across contexts and is a context-specific trait instead.

**Table 1:** Retained variables from the social orienting tests.

Test	Variable	Variable description	*N*
Eye contact	Latency to first gaze	Time elapsed from the moment the last piece of food was given until S establishes eye contact with E	95
	Duration of gaze	Time during which S establishes eye contact with E after receiving the last piece of food	95
Unsolvable task	Relative latency to gaze at human	Time elapsed from the start of the trial until S gazes at a human (E or C) divided by the trial duration for each S	79
	Proportion of attention towards humans	Duration of attention towards a human (gazing/ interacting with E or C) divided by the sum of the duration of attention towards a human and the duration of attention towards the apparatus	79
	Frequency of gaze alternation	Number of times S alternates gaze (gaze at human’s face followed by gaze at apparatus or vice versa, within a 2-second range)	70
	Relative latency to gaze alternation	Time elapsed from the start of the trial until S alternates gaze (gaze at human’s face followed by gaze at apparatus or vice versa, within a 2-second range) divided by the trial duration for each S	70

E = experimenter, S = subject, C = caregiver, N = final sample size.

**Table 2:** Retained variables from the social reward tests.

Test	Variable	Variable description	*N*
Separation episodes	Frequency of approaching C after F	Number of trials they approached C after choosing food	50
Play	Frequency of approaching social toy first	Number of trials S approached the social toy first	78
	Frequency of interacting with social toy first	Number of trials S interacted with the social toy first	60
	Average duration of proximity to E	Average time during which S is in proximity to E across the four trials	77
	Average proportion of social play	Duration of interaction with the social toy divided by the sum of the durations of interaction with both toys (social and individual), averaged across the four trials	58

E = experimenter, S = subject, C = caregiver, N = final sample size.

**Table 3:** Retained variables from the social maintaining tests.

Test	Variable	Variable description	*N*
Synchronization	Movement in phase 1	Whether the S moved in phase 1 after C started moving	85
	Relative synchronization	Duration of synchronized activity (time S and C were simultaneously moving plus time they were simultaneously stationary when C was visible to S) divided by the time C was visible to S	51
Conformity	Proportion of choosing large quantity (test)	Number of trials the S chose the plate with the large quantity of food relative to the total number of trials for each S (in the test)	43
	Change-in-bias	Proportion of trials the S chose the large quantity in the pre-test minus the proportion of trials the S chose the large quantity in the test	43

S = subject, C = caregiver, N = final sample size.

**Table 4:** Significant correlations between social motivation variables.

Variables		*N*	Estimate	*p*-value
Duration of eye contact (EC)	Rel. latency to gaze human (UT)	72	− 0.169	0.039
Rel. latency to gaze human (UT)	Av. duration of proximity (P)	61	*0.183*	0.04
Rel. latency to gaze alternation (UT)	Relative synchronization (SY)	34	− 0.269	0.035
Freq. approach C after F (SE)	Freq. approach social toy first (P)	41	*− 0.326*	0.014
Freq. approach C after F (SE)	Relative synchronization (SY)	25	0.386	0.012
Relative synchronization (SY)	Prop. of trials chose large quantity in test (C)	23	− 0.323	0.043

The test of each variable is indicated in parenthesis. In italics correlations in unexpected direction. C = caregiver; F = food; EC = eye contact test; UT = unsolvable task; P = play test; SE = separation episodes; SY = synchronization; C = conformity.

## Supplementary Information

Below is the link to the electronic supplementary material.

Supplementary Material 1

Supplementary Material 2

## Data Availability

The datasets generated and analysed during the current study are available from the corresponding author on reasonable request.
