# Supplementary material for: Behavioural manifestations of human-directed social motivation in dogs
Source: Sci Rep. 2026 Jan 19;16:4649. doi: 10.1038/s41598-025-34929-w (PMC12868681; doi:10.1038/s41598-025-34929-w)
Supplement: Supplementary file 2 — Supplementary Material 2 [file 41598_2025_34929_MOESM2_ESM.docx]

**Behavioural manifestations of human-directed social motivation in dogs – Supplementary Information**

# Supplementary Methods

## *Detailed descriptions of experimental tasks*

*Eye contact test.* The procedure consisted of two phases of 1 minute each. In the first phase, the experimenter called the subject and started giving them pieces of food from a container out of reach of the animal at variable intervals, while she talked to them (dog-directed speech). A maximum of five pieces of food were delivered. In the second phase, the experimenter called the subject by their name, gave them one final piece of food and started a timer. Then, she gazed at the subject for 1 minute. In the cases in which the subject showed signs of fear with the experimenter standing, the test was carried out with the experimenter crouching down. The caregiver was asked to stay in the same room during the test, and to refrain from looking, petting or talking to the dog.

*Unsolvable task*. The experimenter and the caregiver stood on opposite sides of the apparatus, at approximately 50 cm from it, facing it. The caregiver held the subject, while the experimenter baited the apparatus. The test consisted of three initial solvable trials, in which the experimenter showed the subject how she placed three pieces of food on the lid of the container that was fixed to the board, and then covered them with the plastic container placed upside down. Then, the experimenter took her position and asked the caregiver to release the dog. The caregiver was only allowed to give a command in those cases in which the subject needed it as a signal indicating that they were allowed to eat the food. The trial ended when the subject obtained the food or after 1 minute. If the subject obtained the food in at least two out of three trials, a final trial was carried out, which was unsolvable. In this trial the procedure was the same, with the exception that, after covering the food with the plastic container, the experimenter closed it with the lid that was fixed to the board. The trial ended after 1 minute or if the subject managed to obtain the food. The caregiver was instructed to stay in the designated spot and ignore the subject (avoiding eye contact) during the trial, like the experimenter.

*Separation episodes.* The separation episode started when the subject’s caregiver left home, while the experimenter stayed inside with the subject on leash. The caregiver was instructed to return and enter the house after 1 minute. The caregiver and the experimenter were synchronized and measured the time with a chronometer (generally with their phones). Approximately 45 seconds after the caregiver left, the experimenter showed a piece of food to the subject (their favourite treat), placed it on their food bowl and left the bowl on the floor. The food bowl was placed on a spot opposite to the entrance of the house, so the experimenter and the subject on leash were between the entrance door and the food bowl, which were equidistant. When the minute was over, the caregiver entered the house, closed the door and stood in a spot by the door previously indicated by the experimenter. Then, the experimenter released the subject so they could choose between greeting the caregiver and eating the food. The caregiver was instructed to stand still next to the door without talking to the subject. If the subject approached the caregiver (at arm’s reach), they could greet them as they would usually do. If the subject approached the food bowl, they could eat the treat. If the subject did not make a choice after approximately 1 minute, the trial was ended.

*Play test.* The caregiver was sitting on a chair, holding the subject in front of them. At 2 meters distance there was a plastic tape 1.2 meters long, fixed to the floor with adhesive tape. At the start of each trial, the experimenter stood behind the middle point of the tape and showed two identical toys (rope bone or ball with rope) to the subject, holding one on each hand and shaking them, not looking at the subject. Next, she crouched and placed both toys on the floor simultaneously, one at each end of the tape. Then, she went behind one of the toys and kneeled. After kneeling, the experimenter gave the indication to the caregiver to release the subject. The caregiver was instructed to avoid looking at any toy. The trial lasted 1 minute, during which the subject was free to choose whether to play or not and with which toy, and the caregiver was instructed to remain sitting and ignore the subject. If the subject was playing with the experimenter when the minute ended, the experimenter suddenly let go of the toy, interrupting the game. The interruption period lasted 1 minute, during which the experimenter did not move, only looked at the subject and sometimes took the video camera from her chest to hold it and follow the subjects’ movements. Between subjects, the toys were washed with water and textile detergent not harmful for dogs (in a few cases, usually when several dogs were tested in the same household, the toys presented had not been washed).

*Synchronization test.*  The caregiver and the subject started the test in the same room. The caregiver was sitting on a chair, and once the subject was passive (could be standing, sitting or lying, but not moving), the experimenter indicated the start of the test. Then, the caregiver took a route previously explained by the experimenter. The test consisted of four phases of 15 seconds each, that is, it lasted for 1 minute. Although the time was measured by the caregivers using a chronometer, the duration of the phases turned out to be different for each caregiver, what was considered during the analyses. The experimenter recorded the test with a handheld camera, focusing on the subject, but trying to interfere as less as possible. In addition, a fixed camera was recording in each of the two rooms where the caregiver sat down.

Conformity *test*. The caregiver was sitting on a chair, holding the subject in front of them. At 2 meters distance there was a plastic tape 1.2 meters long, fixed to the floor with adhesive tape. A pre-test was conducted first, in which the subject could choose between two plates with different amounts of food (small amount: one piece; large amount: six pieces). At the start of each trial, the experimenter kneeled behind the middle point of the tape and placed both plates on the floor simultaneously, one on each end of the tape. While she placed the plates the experimenter looked at the subject to check if they looked at both plates. Then, the experimenter stood up and, if the subject had not looked at both plates during placement, she waited until they would do so (with a maximum of approximately 30 seconds). Next, the experimenter turned their back on the subject, took a step forward (away from the tape) and gave the indication for the caregiver to release the subject. The caregiver was instructed to avoid directly looking at the plates and notify the experimenter when the subject started to eat food from one of them, so she could take away the other plate. The pre-test consisted of a maximum of six trials and the goal was to check whether the subject showed a preference for the large amount. Therefore, initially only those subjects that chose the large quantity in at least four trials moved on to the next phase. This criterion was applied to the first 75 subjects, and 47 (63%) did not reach it. Given that a great number of subjects did not reach this criterion, we decided to eliminate it. Thus, from this point on, the pre-test consisted of six trials for all the remaining subjects (n = 24), and all subjects moved on to the following phase independently of their score on the pre-test.

In the test phase, the procedure was similar with the exception that the experimenter showed interest for the plate with the small quantity. After placing both plates on the floor and waiting for the subject to look at both (with a maximum of approximately 30 seconds), the experimenter kneeled behind the plate with the small amount. Then, she grabbed the piece of food, brought it close to her face, looked at it, then at the subject and finally back at the food and said in Spanish with a happy tone “How tasty! How tasty this is!”. Next, she left the piece of food on the plate, stood up to go to the same position as in the pre-test and gave the indication for the caregiver to release the subject.

## *Brief description of other tasks of the battery*

*Sociability test.* The test consisted on an encounter with an unfamiliar human with a passive and an active phase of 1 minute each, based on the protocol in ref.^1^.

*Call test.* In this test, the caregiver called the subject, who was with the experimenter in another room. Six trials were carried out, which were interspersed with the rest of the tests of the first session (i.e., after each test, a trial of the call test was carried out; see **¡Error! No se encuentra el origen de la referencia.**). In half of the trials the caregiver was facing the door (or area) through which the subject would enter the room (*facing* condition), and in the other half they had their back turned to it (*back* condition).

*Object choice task – pointing.* In this test, the subject was presented with two identical opaque cups, one of which was baited. In each trial, the experimenter used dynamic sustained proximal pointing to indicate the baited cup.

*Object choice task – gazing.* The procedure was the same as in the pointing test, with the difference that, instead of pointing with the finger, the experimenter turned her head and gazed at the baited cup.

*Food begging test.* At the start of this test, the experimenter and the subject were facing each other, with a stool between them. After delivering three pieces of food from a container on the stool, the experimenter acted according to the condition of the trial. In the trials of the *back* condition, the experimenter turned 180º, so that she ended with her back turned to the stool with the food container and to the subject. In the trials of the *facing* condition, the experimenter took the stool, placed it behind her and kept her position. Each trial lasted 30 seconds, after which the experimenter turned 180º again (*back* condition) or placed the stool in front of her (*facing* condition). That is, at the end of the trial the experimenter or the stool returned to the starting position. Before the next trial, the experimenter gave one piece of food to the subject to place them again at the starting position.

## *Data pre-processing*

*Separation episodes.* Only subjects that had data for the four trials were considered. Subjects could have missing data in a trial if they did not make a choice or if the trial was not carried out (e.g., due to lack of motivation of the subject or scheduling constraints of the caregiver). The *first choice* variable was used to obtain the number of trials they chose the caregiver first. Given that there was low variability (most subjects approached the food first), this variable was discarded. Since the first choice was the only variable we planned to use initially, at the beginning of data collection the experimenter finished the test immediately after the first choice. However, it was soon clear that this variable would probably not be useful, and that the subjects’ behaviour after their first choice might be more informative. For this reason, we also coded whether the subject approached the option not chosen first (the subjects that were tested first do not have this data since the experimenter did not wait after their first choice). We were especially interested in whether the subjects that chose the food first in the four trials approached and greeted the caregiver afterwards, so we only kept these individuals. For these subjects, we registered the number of trials they approached the caregiver after choosing the food.

*Unsolvable task.* The test was re-coded, considering the test started not when the subject was released, but when the subject first interacted with the apparatus. This meant that each subject had a different test duration and that *latency to interact with apparatus* was dropped. The test duration for each subject was used to calculate relative durations and latencies. For all variables, only subjects that had a test duration ≥ 40 seconds were considered. *Latency to gaze apparatus* was considered irrelevant for the objectives of the present study. *Latency to interact with humans* was eliminated due to low variability (interaction with humans was not frequent, so the majority of subjects had maximum latency). For the *gaze alternations* analyses, we excluded subjects who had gazed at neither the apparatus nor the humans, and included only those who had gazed at one or both. The *duration of gaze* and *duration of interaction* variables were summed to create the variables *duration of attention towards apparatus* and *duration of attention towards humans*. The variable *proportion of attention towards humans* was obtained dividing the *duration of attention towards humans* by the sum of the *duration of attention towards humans* and *towards the apparatus*.

*Play test*. For all variables, only subjects that approached one of the two toys in the four trials were considered. Moreover, for each variable, to avoid having individuals with different number of trials, we kept only those subjects that had data for all trials. Since the *first toy touched* had a low interobserver reliability (on some occasions it was hard to determine whether the subject made physical contact with the toy or only sniffed closely), it was replaced with *first toy interacted with,* which included sniffing, following the toy’s movement with head, and physical contact such as rubbing, nosing, licking, pawing, chewing. This variable had an acceptable kappa value. The *frequency of approaching the social toy first* and the *frequency of interacting with the social toy first* were calculated. The *duration of proximity to E* was averaged across the four trials. The *duration of interaction* with the toys was relative to the time the subject was “visible” (i.e., time during which it could be determined whether the subject was playing with a toy and with which). Only subjects that were “visible” ≥ 40 seconds in the four trials were considered for the variables *duration of interaction with social/individual toy*. For each trial, a *proportion of social play* was calculated by dividing the *duration of interaction with social toy* by the sum of the *duration of interaction with social toy* and the *duration of interaction with individual toy*. The *proportion of social play* was then averaged across the four trials. The interruption period variables were not included in the analyses of this study.

*Synchronization test.* Due to the configuration of the test, the visibility of the caregiver for the subject was a factor that had to be taken into consideration when interpreting the variables. We estimated visibility based on the videos recorded by the three cameras (the camera held by the experimenter plus the fixed camera in each room). In cases where this was unclear, we coded it as non-visible. The *latency* *to switch to the same activity* in each phase was coded as missing if the caregiver was not visible to the subject when changing the activity (i.e., at the start of the phase), or if the subject was already doing the activity to which the caregiver switched (e.g., if the dog was already moving when the caregiver started moving). This resulted in an extremely low sample size for these variables, since on most occasions the caregiver was not visible to the subject at the start of the phase (e.g., the caregiver walked to another room in phase 1 and the dog did not follow him/her, so the caregiver was not visible to the dog when he/she sat down – which was the start of phase 2). Therefore, we decided to keep only the *latency to move in phase 1*, when the subject and caregiver were necessarily in the same room and therefore the caregiver was visible to the subject when starting to move. For ease of interpretation, this variable was transformed into a binary variable, with a value of 1 indicating that the subject moved during phase 1. The *relative synchronization* was calculated as the sum of the *duration of synchronized movement* and *synchronized* *stationary* (i.e., time the caregiver and subject were simultaneously moving/stationary when the caregiver was visible to the subject) divided by the total time the caregiver was visible to the subject. Only subjects for which the caregiver was visible ≥ 40 seconds were considered.

*Conformity test.* Only subjects that completed the test and looked at both plates in all trials of both the pre-test and test were considered. Since the criterion to advance to the test phase was eliminated at a certain point during data collection, some of the subjects that completed the test did not reach this criterion (i.e., not all subjects chose the large amount in 4 trials in the pre-test). The *proportion of trials* *the large amount was chosen* in the pre-test and in the test was calculated for each subject. The number of trials was always 6 for the test phase but varied for the pre-test phase for subjects which were still applied the criterion (they had a maximum of 6 trials to reach criterion but as soon as they reached it the pre-test phase stopped). The criterion of choosing the large quantity in at least 4 out of 6 pre-test trials was eliminated because otherwise the sample size would have been substantially reduced, limiting statistical power. Instead, we calculated a *change-in-bias* score (see also refs.^2,3^) by subtracting the proportion of large-quantity choices in the test from the proportion in the pre-test. This variable was intended to capture the influence of the experimenter’s behaviour on the subjects’ choices during the test (i.e., larger values reflect stronger conformity or social susceptibility).

Nevertheless, the data of this test was affected by an experimenter’s error, which meant that in most trials the plates were not equidistant to the subject. In some cases, it was not possible to determine from the video which of the plates was closer. We performed a binomial logistic regression analysis to determine the effect of the condition, distance, and side of the plate on the subjects’ choices. In this analysis, the dependent variable was whether they chose the plate with the large amount (binary variable), the independent variables were the condition (pre-test or test), the distance of the plate with the large amount (close, far or equidistant, as two dummy variables with equidistant as reference level) and the side of the plate with the large amount (right or left), and the subject was also included as a random effect. First, we tested whether the baseline mixed-effects model (i.e., including random effects) was better than the baseline GLM model (i.e., only fixed effects). Comparison of AICs suggested that including the random effects did not improve the model, so we worked only with the fixed effects. We performed a stepwise comparison, starting by comparing the null model with the model that had condition as a predictor. The model that was significantly better was then tested against a model in which an additional predictor was added. In summary, the model that included condition as a predictor was significantly better than the null model (deviance chi-square test, *χ^2^* = 11.69, *df* = 1, *p* < 0.001), and the addition of the other predictors (i.e., distance, side) did not improve the model (deviance chi-square test*, χ^2^* = 2.01, *df* = 2, *p* = 0.366 and *χ^2^* = 1.29, *df* = 1, *p* = 0.256, respectively). When testing the model with only distance as a predictor against the null model, there was no significant improvement (deviance chi-square test, *χ^2^* = 2.94, *df* = 2, *p* = 0.231). Therefore, it seems that, overall, it was the condition (i.e., pre-test or test) that was driving subjects’ choices.

## *Interobserver reliability*

ICC and kappa values of the relevant variables can be found in Table S3. Some variables showed poor interobserver reliability. The low ICC value for the latency to gaze at humans in the unsolvable task was likely due to differences in the procedure followed when coding the behaviours (continuous versus frame-by-frame or a mix of both) and the time resolution of the coding program. Gazing behaviour was only coded when the subject was stationary, but there were instances in which the perception of whether the subject was stationary or moving changed depending on whether the video was coded continuously or frame-by-frame. This issue also affected the variables frequency of and latency to gaze alternation to a degree. The variable *first toy touched* had a low kappa value and was then replaced by the variable *first toy interacted with*, which showed acceptable interobserver reliability.

| **Test** | **Variable** | **Estimate (95% CI)** | **Coefficient** |
| --- | --- | --- | --- |
| Eye contact | Latency to first gaze | .99 (.96 – .99) | ICC |
|  | Duration of gaze | .99 (.98 – 1.00) | ICC |
| Separation episodes | Approaches C after choosing F | .87 | Kappa |
| Unsolvable task | Latency to gaze human | *.53 (.05 – .81)* | ICC |
|  | Duration of gaze towards apparatus | .99 (.97 – 1.00) | ICC |
|  | Duration of gaze towards humans | .98 (.94 – 1.00) | ICC |
|  | Duration of interaction with apparatus | .98 (.53 – 1.00) | ICC |
|  | Frequency of gaze alternation | *.64 (.26 – .86)* | ICC |
|  | Latency to gaze alternation | *.65 (.25 – .86)* | ICC |
| Play | First toy approached | .92 | Kappa |
|  | First toy interacted with | .69 | Kappa |
|  | Duration of proximity to E | .99 (.97 – .99) | ICC |
|  | Duration of interaction with “social” toy | .95 (.86 – .98) | ICC |
|  | Duration of interaction with “individual” toy | .91 (.86 – .94) | ICC |
| Synchronization | Duration of synchronized movement | .99 (.97 – 1.00) | ICC |
|  | Duration of synchronized stationary | .90 (.79 – .96) | ICC |
|  | Latency to move phase 1 | .94 (.85 – .98) | ICC |

**Table S4.** *Interobserver reliability.* In italics are values below acceptable levels (estimate values: ICC < .7; Kappa < .6). E = experimenter; S = subject; C = caregiver

# References

1. Jakovcevic, A., Mustaca, A. & Bentosela, M. Do more sociable dogs gaze longer to the human face than less sociable ones? *Behav. Processes* **90**, 217–222 (2012).

2. Turcsán, B. *et al.* Personality traits in companion dogs—Results from the VIDOPET. *PLoS One* **13**, (2018).

3. Kis, A., Bolló, H., Gergely, A. & Topál, J. Social Stimulation by the Owner Increases Dogs’ (Canis familiaris) Social Susceptibility in a Food Choice Task - The Possible Effect of Endogenous Oxytocin Release. *Animals* **12**, 296 (2022).
